# Prevalence of Malnutrition in Orally and Tube-Fed Elderly Nursing Home Residents in Germany and Its Relation to Health Complaints and Dietary Intake

**DOI:** 10.1155/2011/247315

**Published:** 2011-05-19

**Authors:** Dorothee Volkert, Lioba Pauly, Peter Stehle, Cornel C. Sieber

**Affiliations:** ^1^Institute for Biomedicine of Aging, University of Erlangen-Nürnberg, Heimerichstraße 58, 90419 Nuremberg, Germany; ^2^Institute of Nutrition and Food Sciences, Nutrition Physiology, University of Bonn, 53115 Bonn, Germany

## Abstract

*Objective*. To investigate the prevalence of malnutrition in orally and tube-fed nursing home (NH) residents in Germany and its relation to common health complaints and dietary intake. *Methods*. In 350 NH residents, subjects' characteristics, Mini Nutritional Assessment (MNA), and several health problems were inquired with the nursing staff using standardised interviews. In a subset of 122 residents, dietary intake was assessed by 3-day weighing records. *Results*. 7.7% of the participants were tube fed. 24.1% of orally nourished and 57.7% of tube-fed residents were malnourished (MNA < 17 p.). Malnutrition was significantly related to nausea/vomiting, constipation, pressure ulcers, dehydration, infections, antibiotic use, and hospitalisation. Mean daily energy intake was 1535 ± 413 kcal and mean protein intake was 54.2 ± 0.9 g/d irrespective of the nutritional state. *Conclusion*. In Germany, malnutrition is widespread among NH residents and is related to common health problems. The MNA rather reflects health condition than currently reduced dietary intake.

## 1. Introduction

Elderly people are at increased risk of malnutrition due to a variety of factors including sensory losses, loss of appetite, chewing and swallowing problems, mobility restrictions, cognitive impairment and depressive mood, acute and chronic diseases, and accompanying multimedication [1]. Due to the frequently reduced physical and mental functioning, nursing home (NH) residents are particularly affected. In previous studies, however, highly differing prevalence rates of malnutrition were reported in institutionalized elderly. In a recent literature review about the nutritional situation of elderly nursing home residents, we found a reduced body-mass index (BMI < 20 kg/m²) in 10 to 50% of the residents studied; weight loss was reported with prevalence rates between 5 and 41%. According to the Mini Nutritional Assessment (MNA), malnutrition was observed in 2 to 38% and a risk of malnutrition in 37 to 62% [2]. In an Italian [3] and a Swedish [4] study, even 71% of NH residents were found to be malnourished. In Germany, presently about 700,000 elderly are living in institutions and, as in many other countries, an increase is expected due to demographic changes [5]. Nutritional status of this growing population group has not thoroughly been studied before in Germany. 

Generally, malnutrition is caused by an ongoing insufficient intake of energy and nutrients. In order to prevent malnutrition in persons who are persistently unable to eat adequate amounts of food, enteral nutrition by means of tube-feeding can be applied. Nutrition via a percutaneous endoscopic gastrostomy (PEG) is an established method for long-term enteral nutrition and is often used in nursing home residents not able to eat adequate amounts of food, although not without controversy [6, 7]. One of the main reasons of controversy may be the fact that enteral nutrition may be used incorrectly to facilitate care or save time instead of spending attention and time to oral feeding. Based on a recent nation-wide mailing survey, it was estimated that about 40,000 NH residents in Germany are living with a PEG [8]. Our present knowledge about nutritional and health status of nursing home residents who are tube-fed is poor. 

Tube-feeding often goes along with gastrointestinal (GI) complaints like nausea and vomiting, constipation or diarrhoea [9], but such symptoms are also frequently reported in orally nourished elderly and may compromise adequate dietary intake and contribute to the risk of malnutrition [1]. On the other hand, malnutrition increases the risk of illness, for example, infections, and may worsen the course of acute and chronic diseases. This association has mainly been reported in the acute-care setting for geriatric patients [10–13]. Little is known about health complaints of nursing home residents and the relation between malnutrition, common health complaints, and dietary intake.

Thus, the *aim* of the present study was to evaluate the prevalence of malnutrition in orally and tube-fed elderly nursing home residents in Germany and the relation between malnutrition, health problems, and dietary intake. We hypothesized that malnutrition and health complaints are widespread and interrelated, and that dietary intake is markedly reduced in malnourished residents.

## 2. Methods

### 2.1. Study Design

In this cross-sectional study, all residents from 3 municipal nursing homes (NHs) in Bonn, Germany, were considered for inclusion if they were at least 65 years old, in long-term care, and not in a terminal state (judged subjectively by the responsible nurse). Subjects' characteristics, nutritional status, health complaints, and dietary intake were assessed once in each participant between November 2004 and April 2006. The study was approved by the local ethics committee, and all participating subjects gave a signed consent.

### 2.2. Subjects' Characteristics

Subjects' characteristics were assessed in standardised personal interviews with the responsible qualified nurse, and included date of birth, gender, length of stay in the nursing home, route of feeding (oral or tube fed), and the following physical and mental aspects. The ability to perform basic *activities of daily living* (ADL) was assessed according to Mahoney and Barthel [14]. Residents were classified as independent (>65 p.), in need of help (35–65 p.), and in need of care (<35 p.). Residents were classified as *mobile* if they were able to walk at least 50 m without personal help, as partly mobile if they were able to walk at least 50 m with help or move independently with a wheel chair, or as immobile if they were unable to move at least 50 m. Kind and number of *chronic diseases* and of prescribed *medications* were gathered from the medical folders. The participants' general *health status* was subjectively judged by the nursing staff as fair, moderate, or poor. *Mental status* (no, mild, severe dementia; no, mild, severe depression) was also rated by the nursing staff by clinical judgment. In tube-fed residents, date of tube-placement and reason for tube-feeding were asked as well as the daily amount of tube-feed and additional oral food intake (no, little, predominant).

### 2.3. Nutritional Status

The *Mini Nutritional Assessment *(MNA) was used for the assessment of malnutrition. This standardized questionnaire, specifically designed for the elderly, consists of 18 questions with given weighted answers that sum up to a maximum score of 30 points. Patients are classified as well nourished (≥24 p.), at risk of malnutrition (17–23.5 p.), or malnourished (<17 p.) [15, 16]. In tube-fed residents, MNA questions concerning anorexia (A) and quality of diet (J–M) were scored highest, assuming an adequate provision of nutrients due to nutritionally complete tube-feeds and supposed specific nutritional attention for these residents.

*Body mass index *(BMI) was calculated as weight/(height²) based on measured weight and height. Residents were weighed with a digital chair scale (Seca, Hamburg, Germany) to the nearest 0.1 kg. Height was measured with a measuring rod to the nearest 0.1 cm with the resident standing without shoes. When patients where unable to stand or had either deformations of the spinal column or osteoporosis, knee height was measured to the nearest 0.1 cm and height calculated according to Chumlea et al. [17]. The prevalence of BMI values below 20 and below 22 kg/m² was calculated.

*Midarm circumference *(MAC) was measured at the midpoint of the relaxed, nondominant arm between the tip of the acromion and the olecranon process. 

*Calf circumference *(CC) was measured at the widest part of the undressed calf. 

Both measurements were performed with a plastic tape measure and an accuracy of 0.1 cm and were utilised for the anthropometric questions in the MNA. Values below 21 cm (MAC) and below 31 cm (CC) were considered as reduced, respectively.

### 2.4. Health Complaints

The presence of nausea/vomiting, constipation, diarrhoea, pressure sores, wound healing problems, and dehydration was assessed in a standardised manner by interviewing the responsible nurse. The frequency of infections, antibiotic treatment, and hospitalisation in the previous three months was collected from the medical folders in cooperation with the responsible nurse.

### 2.5. Dietary Intake

In a subgroup of 122 orally fed residents, dietary intake was monitored for three consecutive days by precisely weighing all offered food before and all leftovers after each meal, using a digital weighing machine. Due to the high work load related to this method, dietary assessment was restricted to the residents of two nursing units of each of the 3 nursing homes. Foods were coded and analyzed for nutrient composition using the German nutrient database (BLS II.3) [18]. The mean intake of energy and protein was calculated per day and per kg body weight. 

All measurements and assessments were performed by the same trained person (LP).

### 2.6. Evaluation and Statistics

Data analysis was performed using SPSS version 17.0 (SPSS Software, Munich, Germany). Categorical variables are reported as absolute numbers and percentages. Continuous variables are presented as mean ± standard deviation (SD), median, and 25th and 75th percentiles (P_25_–P_75_). Subjects' characteristics and prevalence rates of malnutrition and of health complaints are reported in orally and tube-fed residents; the prevalence of health complaints and dietary intake is reported according to the MNA groups. Chi-square testing was used to detect differences between categorical variables. The normal distribution of continuous variables was tested by Kolmogorov-Smirnov test. Differences in continuous variables between subgroups are analysed by *t*-test or ANOVA and Tukey's post hoc test if normally distributed. Otherwise, Mann-Whitney-*U* and Kruskal-Wallis test were used. Missing values were not considered for statistical analysis. For all tests, *P* values below  .05 were considered statistically significant.

## 3. Results

### 3.1. Subjects' Characteristics

Out of 382 persons residing in the institutions, 15 had to be excluded. Nine were younger than 65 years, four in a terminal state, and three in short-term care; one person was permanently hospitalized, one removed, and one deceased before data collection. 13 residents refused to participate. 350 residents agreed to take part, 283 women and 67 men with a mean age of 84.8 ± 8.0 years. The median length of stay in the institution was 2.7 years (1.3–4.9 years). 

27 residents (7.7%) had a PEG *in situ*. About half of them (*n* = 15) were fed completely via this route and received either 1500 mL (*n* = 8) or 1000 mL (*n* = 7) per day of a standard tube-feed. Four residents were predominantly tube fed (mean 938 mL/day), and seven residents mainly received oral food and some tube-feed in addition (mean 431 mL/d). One resident received only water via the PEG. The reasons for tube-feeding were dysphagia (*n* = 13), refusal to eat (*n* = 5), low food (*n* = 3) or fluid (*n* = 3) intake, in one case a tumor and in one case an oesophagitis. In one case, the reason was unclear. 25 residents were fed continuously and two per bolus. The median duration of tube-feeding was 17.9 months (5.6–26.5 months). Ten residents already had the PEG when they moved to the NH, five received it within one year, one after one year and 11 after more than two years of residence in the NH.

All together, a considerable proportion of the residents were disabled. About one-third was in need of care (37.4%), immobile (34.9%), and/or showed signs of depression (38.0%), respectively. In nearly two-thirds (61.4%), signs of dementia were reported. Most of the participants (55.0%) suffered from 5 or more chronic diseases and nearly three-fourths (70.9%) took 5 or more prescribed medications. Nevertheless, health status of 83.1% of the study population was judged as fair or moderate. The most prevalent medical diagnoses were hypertension (43.3%), dementia (39.8%, as routinely documented by a practitioner), and cardiac insufficiency (32.1%). Diabetes mellitus was prevalent in 24.4%, osteoporosis in 15.8%, and kidney disease in 9.2%. 13.2% suffered from a previous stroke, 7.4% from a tumor and 6.3% from respiratory disease.

The subjects' characteristics are shown in Table 1 for orally and tube-fed residents separately. Tube-fed residents were significantly more often care dependent, immobile, severely demented, and in a poor health state than residents with oral nutrition. There was no difference in the number of chronic diseases or medications and no difference in the prevalence of specific diseases except stroke, which was reported in nearly half of the tube-fed subjects (48.1%), but only in 10.2% of orally nourished residents (*P* < .001). 

### 3.2. Nutritional Status

According to the MNA, more than one-forth (26.7%) of the total group suffered from malnutrition (MNA < 17 p.) and one-half (52.9%) was at risk (MNA 17–23.5 p.). Malnutrition was significantly more prevalent in tube-fed compared to orally nourished residents (*P* < .001; Table 2). The mean BMI was 25.5 ± 5.1 kg/m² (22.0–28.2 kg/m²; *n* = 334) without difference between orally and tube-fed residents. In 13.5%, the BMI was below 20 kg/m², and 25.1% had a BMI below 22 kg/m². MAC was reduced in 12.9%, again without significant difference between tube- and orally nourished residents. CC was reduced in half of the orally nourished (50.2%) and in three-quarts (76.9%) of the tube-fed residents (*P* < .001).

### 3.3. Health Complaints

**Figure 1:**
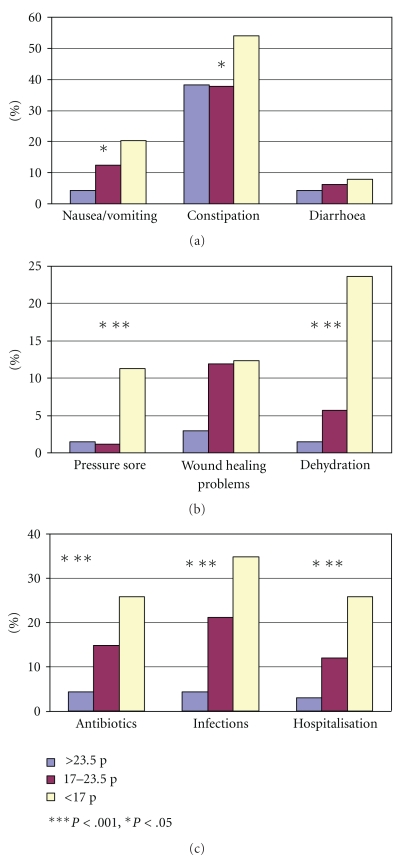
Prevalence of health complaints in well-nourished residents (MNA > 23.5 p.; *n* = 68), residents at risk (MNA 17–23.5 p.; *n* = 176), and malnourished residents (MNA < 17 p.; *n* = 89).

Constipation was reported in 43.0% of all residents, nausea/vomiting in 13.4%, and dehydration and wound healing problems in 10.6%, respectively. Diarrhoea (6.3%) and pressure sores (3.7%) were less frequent. Constipation and nausea/vomiting were significantly more frequent in tube-fed residents (Table 3). Within the previous three months, 22.3% had an infection, in 16.3% treated with antibiotics, and 14.9% were hospitalised without difference between orally and tube-fed residents. All health problems except diarrhoea and wound healing problems were significantly more often reported in malnourished residents (Figure 1).

### 3.4. Dietary Intake

The weighing records revealed a mean daily energy intake of 1535 ± 413 kcal (6.42 ± 1.72 MJ) and a protein intake of 54.2 ± 0.9 g/d. Expressed per kg BW the residents consumed 25.5 ± 7.3 kcal and 0.89 ± 0.27 g protein. Dietary intake according to MNA is presented in Table 4. There was no difference between the groups in total intake of energy and protein per day. Expressed per kg BW, both energy (*P* < .001) and protein (*P* < .05) intake were significantly higher in malnourished residents compared to well-nourished ones. The difference in energy intake per kg BW between malnourished and those at risk of malnutrition was also significant (*P* < .05).

## 4. Discussion

In this cross-sectional study, nutritional status was studied for the first time in a large sample of nursing home residents in Germany. A considerable proportion of the residents were found to be malnourished or at risk of malnutrition. 

Prior to that, only two smaller studies addressed the nutritional situation of institutionalized elderly in Germany. One was restricted to 50 apparently healthy women living in two old peoples' homes and reported a generally good nutritional status [19]. The other focused on dietary intake and physical activity of 47 female self-feeding and 20 eating-dependent NH residents [20]. Meanwhile, two large-scale studies with more than 2000 participants in each study were performed—one in 29 German [21], the other one in 30 German and 8 Austrian nursing homes (“nutritionDay”) [22]. In both projects, questionnaire-based assessments were performed by local nurses at one specific day. Another regional study recently looked at nutritional and functional status of 200 NH residents in Nuremberg [23, 24]. 

One strength of the present study is its high participation rate, meaning that the results are representative for the participating institutions. This could be achieved mainly because participation was strongly recommended and supported by the nursing home management. In addition, all information except four anthropometric measurements was collected in cooperation with the nursing staff, implying only minimal burden for the participants. Detailed data were assessed in personal interviews with the responsible nurses. These interviews were scheduled on office days destined for documentation. Thus, enough time was available despite usually high work loads for nursing staff. All nurses were familiar with their dedicated residents and well informed about their personal characteristics and health situation, so that reliable information could be obtained. For the MNA, it has recently been reported that application by the nursing staff is even superior to direct interviews with the residents, because more complete and detailed, and, especially in demented subjects, more reliable information can be obtained [23]. Nevertheless, it has to be mentioned that categorization of some parameters, especially dementia, depression, and general health status, is affected by subjective perceptions. More detailed assessments were intentionally abandoned in order to keep the burden for the residents as well as total expenses low. 

As characteristic for the nursing home population, considerable proportions of the residents were physically and mentally impaired with multiple chronic diseases, multimedication, and a reduced level of self-sufficiency (Table 1). Very similar rates of immobility (30%) and dementia (68%) were reported by Valentini et al. [22] in the above-mentioned “nutritionDay” study from 30 German and 8 Austrian NHs. 

Regarding dementia, different prevalence rates are noticeable according to the nurses' perception (61.4%) and the diagnosis found in the medical records, routinely documented by a practitioner (39.8%). Presumably, the prevalence was underestimated by physicians, who often miss to diagnose mental impairments [25], and, on the other hand, overestimated by the nursing staff, who might have wrongly interpreted acute or other forms of cognitive impairment (e.g., delirium) as dementia. With respect to malnutrition, however, also mild forms of confusion may be relevant.

As currently recommended [26], malnutrition was assessed by using the MNA, a simple and well-validated instrument, especially designed for the elderly and regarded as the gold standard for nutritional assessment for elderly living in long-term care facilities [27]. The strength of the MNA lies in its multidimensional approach which comprises physical and mental state, health, and self-perception, as well as nutritional status and quality of the diet. The risk of malnutrition may be detected early with this tool, and preventive measures initiated in a timely manner. According to the MNA, we found malnutrition in about one quarter of the residents, and more than half were at risk of malnutrition. Compared to international data, these prevalence rates are in the middle of previously reported ranges [2].

Regarding BMI, about one quarter of the residents showed values below 22 kg/m², and a BMI below 20 kg/m² was observed in 13.5%—somewhat less frequent than reported in other nursing home populations [22, 23, 28–32]. 

CC was reduced in more than half of the residents (52.2%). This clearly indicates reduced leg muscle mass and protein stores, caused by disuse of the leg muscles, and reflects the low mobility level in our population. In community-living elderly, a CC below 31 cm was identified as best clinical marker of sarcopenia and associated with disability and reduced leg function [33]. Up to now, CC was measured only in a few studies, all reporting higher mean values [23, 34–36]. Ruiz-López et al. [35] observed reduced values (<31 cm) in 30% of 89 NH residents in Spain. Unfortunately, in all these studies, mobility or activity level were not reported. Importantly, CC was much more often reduced than MAC (13%). All residents with reduced MAC, except one, also had a reduced CC. This difference may be explained by less pronounced muscle mass in upper extremities and less pronounced changes as a result of inactivity. 

In tube-fed residents, complete ADL dependence and the high prevalence of immobility and dementia are striking (Table 1). There was no difference in kind and number of chronic diseases, except for stroke, one of the main indications for tube-feeding. However, health status was more often judged to be poor, and these persons likely are in rather advanced disease states. 

Regarding tube-feeding of NH residents, there is an ongoing discussion about its benefits and risks. Especially in case of severe dementia the benefit of enteral nutrition is questioned [6]. In our participants, the adequacy of this mode of feeding cannot be judged, since decisions concerning tube-feeding are always very individual—depending on the patients' underlying disease, general condition, and personal preferences. Stated reasons for tube-feeding were dysphagia and low food or fluid intake in most of the cases and, thus, appropriate indications for enteral nutrition [37]. Compared to the recent nation-wide survey of Wirth et al. [8], where 6.6% of all residents of the responding nursing homes were fed via PEG, the prevalence of tube-feeding in our population was very similar. Additional food intake was slightly more common compared to Wirth et al. [8] (44 versus 36%), and a slightly smaller proportion (37 versus 50%) received the PEG before NH admission.

Despite scoring the highest for the five questions regarding appetite and diet quality, a low total MNA score and, thus, a high prevalence of malnutrition were observed in tube-fed residents. This is in line with a number of earlier studies, which have reported a reduced nutritional status in elderly patients at the time of tube placement. These studies, however, referred to low BMI and albumin values [38–42]. In our population, however, BMI was not different in orally and tube-fed residents, and markedly higher than in these studies. Also, MAC did not differ between the two groups (Table 2). These results demonstrate that a normal body mass index can be maintained or achieved by tube-feeding, that is not reflected by the results of the MNA. CC, in contrast, was significantly lower and more often reduced in tube-fed compared to orally nourished residents (Table 2). This reflects the higher proportion of immobility in these subjects and shows that nutritional support alone, without concomitant physical activity, is not effective in improving muscle mass and function.

Gastrointestinal disorders, common in the elderly, may result in complications and can cause major morbidity [43, 44]. With respect to nutrition, they may negatively affect dietary intake and compromise nutritional status.

In our study, *constipation* was by far the most prevalent health complaint—nearly half of our participants were affected. An approximately equivalent prevalence rate was reported in a large Finish NH population [45]. Constipation is favored by age-related changes in gastrointestinal function, for example, weakening of the colonic muscles and changes in anorectal function [43, 46]. It also occurs as an adverse effect of many medications. Thus, the high prevalence may partly be explained by the observed multimedication in our study. Nutritional factors as decreased food, fluid, and fiber intake may also contribute to its development. In our orally nourished participants, with a mean of about 1500 kcal/d food intake was often rather low. Mean fiber intake from food amounted to 12.8 g/d, and thus was also clearly below the recommendation. In only 8 of the 27 tube-fed residents, a fiber-containing feed was used. On the other hand, constipation may lead to discomfort, feeling of fullness, and reduced desire to eat and thus, promoting malnutrition. In a recent Swedish study among older adults in sheltered housing, constipation was identified as one of the strongest risk factors for underweight and weight loss [47]. In agreement and corroborated by Suominen et al. [45], constipation was significantly correlated to malnutrition in our study (Figure 1). 

All other health complaints were much less common. *Nausea and vomiting* were reported in 13%. Like constipation, these complaints were more prevalent in tube-fed than in orally nourished residents (Table 3) and significantly related to malnutrition (Figure 1). In contrast, *diarrhoea* was only occasionally reported and neither related to feeding mode nor to malnutrition. Only one tube-fed resident suffered from diarrhea. Obviously, this typical complication of enteral nutrition is avoidable by experienced care. Despite poorer general health, also the other health complaints were not more often observed in tube-fed residents (Table 3); dehydration even tended to be less frequent.

The prevalence of *pressure ulcers* tended to be higher in tube-fed residents, but altogether was very low, despite a high prevalence of immobility and great proportion of bedridden residents in our study—indicating a high quality of care also in this respect. Markedly higher prevalence rates were reported in the above-mentioned German large-scale studies [21, 22]. In accordance with our results, in both of these studies, a close relationship between malnutrition and pressure ulcers is reported, confirming malnutrition as risk factor for this serious health problem [48].

Infections, antibiotic use, and hospitalizations were relatively common in our study population (15–20%; Table 3) and also clearly associated with malnutrition (Figure 1). This close correlation may partly be explained by the fact that one question of the MNA (question D) asks for acute disease in the past three months and, thus, some overlap in this regard must be admitted. 

Interestingly, BMI (neither <20 nor <22 kg/m²) was not related to health complaints (with the exception of dehydration that was significantly more frequent in subjects with reduced BMI; data not presented), suggesting that the MNA reflects general health condition better than the BMI, again strengthening its usefulness in multimorbid geriatric persons.

Mean dietary intake, assessed by precise weighing of all food for three consecutive days in a subgroup of 122 residents, was 1535 kcal and 54 g protein per day. In several studies in recent years, very similar figures were reported for NH residents [35, 49–52]. This amount of energy is clearly below the recommended amount for healthy elderly [53]; however, its adequacy is difficult to estimate, due to limited knowledge about the exact requirements in this very old, multimorbid, mainly disabled persons. Based on body weight, height, and age, a mean basal metabolic rate (BMR) of 1243 ± 170 kcal was calculated in our population, and an energy intake/BMR ratio of 1.24 ± 0.31. This level is regarded as adequate for immobile elderly, but probably is too low for more active persons. The observed mean protein intake of 0.89 g/kg in fact meets the current recommendation for healthy elderly; however, higher protein needs are suggested for frail and multimorbid elderly. Additional offers of milk-based snacks, food fortification, or nutritional supplements might contribute to improve intake of protein as well as energy and other nutrients.

Unfortunately, nutrient intake of tube-fed residents was not assessed in detail in our study. Those residents who were fed completely by tube received either 1 or 1.5 L of a standard tube-feed, and thus, had at least a basic supply of energy and all essential nutrients. Again, adequacy is difficult to estimate because requirements are not exactly known.

In contrast to our expectations and in contrast to Vellas et al. [54] who reported close correlations between the MNA and dietary intake in 105 geriatric patients and 50 community-living elderly, we found no difference in energy and protein intake between residents with malnutrition, at risk of malnutrition, or without malnutrition. Per kg BW malnourished subjects consumed even more energy and protein than those in better nutritional status (Table 4). Ruiz-López et al. [35] also reported a significantly lower energy intake in 5 malnourished NH residents compared to 56 subjects at risk of malnutrition; however, in accordance with our study, no difference between the MNA groups was found for protein intake per day and per kg BW. Ödlund Olin et al. [52] observed in 80 elderly service flat residents and Murphy et al. [55] in 49 female elderly orthopedic patients no difference in energy intake between the MNA groups. These results suggest that malnutrition evolved from a poor intake in the past, possibly caused by an acute event. After relief of the acute problem, intake may normalize without regaining a well-nourished state. This is consistent with a reported increased energy requirement per kg BW in malnourished compared to normally nourished elderly [56]. 

In conclusion, malnutrition is widespread among nursing home residents also in Germany and related to common health complaints but not to currently reduced dietary intake. According to the MNA, enterally nourished residents are markedly more often affected by malnutrition than orally nourished residents. On the other hand, our data show that a normal body mass can be maintained or achieved by tube-feeding, indicated by BMI and MAC, that is not reflected by the results of the MNA. Our data suggest that the MNA rather reflects general condition and nutritional risk than current body stores or dietary intake of energy and protein.

## Figures and Tables

**Table 1:** Characteristics of orally and tube-fed nursing home residents.

	Oral nutrition (*n* = 323)	Tube-feeding (*n* = 27)
Female sex	81.4	74.1
Age, years (mean ± SD (median))	85.0 ± 8.1 (86.0)	81.9 ± 6.2 (82.0)
Age ≥ 85 years (%)	55.1	40.7
ADL, p. (median (P_25_–P_75_))	55 (20–85)	0 (0–5) ***
ADL		
Independent (70–100 p.) (%)	41.8	0.0 ***
In need of help (35–65 p.) (%)	26.0	0.0
In need of care (<35 p.) (%)	32.2	100.0
Mobility		
Mobile (%)	59.4	0.0 ***
Moderately mobile (%)	10.8	3.7
Immobile (%)	29.7	96.3
Dementia		
No [%]	40.2	15.4 **
Mild [%]	20.1	11.5
Severe [%]	39.6	73.1
Depression		
No (%)	61.4	69.2
Mild (%)	22.1	7.7
Severe (%)	16.5	23.1
Health status		
Fair (%)	58.2	25.9 ***
Moderate (%)	27.2	29.6
Poor (%)	14.6	44.4
No. of chronic diseases (median (P_25_–P_75_))	5 (3–6)	5 (4–7)
≥5 chronic diseases (%)	53.7	70.4
No. of medications (median (P_25_–P_75_))	6 (4–8)	5 (4–8)
≥5 medications (%)	70.9	70.4

***P < .001, **P < .01.

ADL = Activities of daily living, SD = standard deviation, P = percentile.

**Table 2:** Nutritional status of orally and tube-fed nursing home residents.

	Oral nutrition (*n* = 323)	Tube-feeding (*n* = 27)
	mean ± SD (*n*)	mean ± SD (*n*)
	median (P_25_–P_75_)	median (P_25_–P_75_)
MNA (p.)	19.9 ± 4.6 (307)	16.0 ± 2.7 (26)
	20.5 (17.0–23.0)	16.0 (14.0–18.0) ***
MNA		
<17 p. (%)	24.1	57.7 ***
17–23.5 p. (%)	53.7	42.3
>23.5 p. (%)	22.1	0.0
BMI (kg/m²)	25.6 ± 5.2 (308)	24.9 ± 4.9 (26)
	25.3 (22.0–28.4)	25.0 (22.0–28.4)
BMI <20 kg/m² (%)	13.6	11.5
BMI <22 kg/m² (%)	25.3	23.1
MAC (cm)	25.3 ± 3.9 (315)	24.8 ± 4.2 (27)
	24.8 (22.9–27.6)	24.9 (22.4–27.8)
MAC <21 cm (%)	12.7	14.8
CC (cm)	31.2 ± 4.8 (315)	27.4 ± 4.5 (26) ***
	30.9 (28.0–34.2)	27.8 (25.3–29.8)
CC <31 cm (%)	50.2	76.9 **

***P < .001, **P < .01.

MNA = Mini Nutritional Assessment, p. = points, BMI = body mass index, MAC = midarm circumference, CC = calf circumference, SD = standard deviation.

**Table 3:** Health complaints of orally and tube-fed nursing home residents.

	Oral nutrition		Tube-feeding	
	(*n* = 323)		(*n* = 27)	
	*n*	%	*n*	%
Constipation	133	41.3	17	63.0 *
Nausea/vomiting	38	11.8	9	33.3 **
Diarrhoea	21	6.5	1	3.7
Pressure sore	8	2.5	5	18.5
Wound healing problems	34	10.5	3	11.1
Dehydration	36	11.1	1	3.7
Infection	70	21.7	8	29.6
Antibiotic use	51	15.8	6	22.2
Hospitalization	45	13.9	7	25.9

**P < .001, *P < .01.

**Table 4:** Dietary intake in nursing home residents with malnutrition, at risk of malnutrition and without malnutrition.

	Well-nourished MNA > 23.5 p. (*n* = 25)	At risk MNA 17–23.5 p. (*n* = 56)	Malnourished MNA < 17 p. (*n* = 41)
Energy (kcal/d)	1516.5 ± 431.2	1566.7 ± 420.2	1502.8 ± 398.0
Energy (kcal/kg BW)	21.9 ± 5.3	24.3 ± 7.0	29.3 ± 7.4 ^∗∗∗,§§^
Protein (g/d)	56.4 ± 20.3	56.0 ± 19.0	50.4 ± 14.8
Protein (g/kg BW)	0.81 ± 0.23	0.86 ± 0.27	0.98 ± 0.28 *

***P < .001, *P < .01 malnourished versus well-nourished.

^§§^ *P* < .01 malnourished versus at risk of malnutrition.

MNA = Mini Nutritional Assessment, p. = points, BW = body weight.
